# Synergistic Gene Expression Signature Observed in TK6 Cells upon Co-Exposure to UVC-Irradiation and Protein Kinase C-Activating Tumor Promoters

**DOI:** 10.1371/journal.pone.0139850

**Published:** 2015-10-02

**Authors:** Kyle P. Glover, Zhongqiang Chen, Lauren K. Markell, Xing Han

**Affiliations:** 1 DuPont Haskell Global Centers for Health & Environmental Sciences, Newark, Delaware, United States of America; 2 Department of Biological Sciences, Cell and Molecular Biology Graduate Program, University of the Sciences, Philadelphia, Pennsylvania, United States of America; 3 DuPont Industrial Biosciences, Wilmington, Delaware, United States of America; CSIR-Indian Institute of Toxicology Research, INDIA

## Abstract

Activation of stress response pathways in the tumor microenvironment can promote the development of cancer. However, little is known about the synergistic tumor promoting effects of stress response pathways simultaneously induced in the tumor microenvironment. Therefore, the purpose of this study was to establish gene expression signatures representing the interaction of pathways deregulated by tumor promoting agents and pathways induced by DNA damage. Human lymphoblastoid TK6 cells were pretreated with the protein kinase C activating tumor promoter 12-O-tetradecanoylphorbol-13-acetate (TPA) and exposed to UVC-irradiation. The time and dose-responsive effects of the co-treatment were captured with RNA-sequencing (RNA-seq) in two separate experiments. TK6 cells exposed to both TPA and UVC had significantly more genes differentially regulated than the theoretical sum of genes induced by either stress alone, thus indicating a synergistic effect on global gene expression patterns. Further analysis revealed that TPA+UVC co-exposure caused synergistic perturbation of specific genes associated with p53, AP-1 and inflammatory pathways important in carcinogenesis. The 17 gene signature derived from this model was confirmed with other PKC-activating tumor promoters including phorbol-12,13-dibutyrate, sapintoxin D, mezerein, (-)-Indolactam V and resiniferonol 9,13,14-ortho-phenylacetate (ROPA) with quantitative real-time PCR (QPCR). Here we show a novel gene signature that may represent a synergistic interaction in the tumor microenvironment that is relevant to the mechanisms of chemical induced tumor promotion.

## Introduction

Cancer cells are characterized by altered signaling programs, genomic instability and dedifferentiation [1]. These characteristics are acquired through a multistage process in which cells selectively become resistant to growth regulation and develop progressively more aberrant growth patterns. In the multistage mouse model, tumor promoters such as 12-O-tetradecanoyl-phorbol-13-acetate (TPA) enhance the development of H-Ras transformed cells by causing altered protein kinase C (PKC) signaling, sustained inflammation, regenerative hyperplasia and oxidative stress [2, 3]. The TPA induced tumor microenvironment thus promotes the development of malignant traits as precancerous cells adapt to adverse growth conditions and acquire a survival advantage [1, 4]. Sustained exposure to these conditions is required since tumor promotion by TPA is a reversible process that requires repeated treatments to maintain the tumor promoting microenvironment [2]. Cells exposed to this sustained pressure must tolerate the many pleiotropic effects of tumor promoter exposure on downstream signal transduction pathways, such as the protein kinase C pathway or interference with other stress response pathways important in carcinogenesis.

A major pathway affected by PKC-activating tumor promoters is the DNA damage response (DDR). TPA has previously been shown to alter the cellular response to DNA damage in various *in vitro* or *in vivo* models [5–10]. Considering that the DDR is constitutively activated in early tumors in response to oncogenic signaling and uncontrolled DNA replication, interaction between tumor promotor altered stress response pathways and the DDR is likely to occur [11, 12]. We have previously shown that tumor promoter pretreated TK6 cells become hypersensitive to DNA damage induced by UVC-irradiation and undergo a synergistic increase in apoptosis, delayed DNA repair and have altered expression of p53-target genes [13]. However, there remains limited knowledge about the synergistic effects of tumor promoters on DDR signaling and whether or not these synergistic effects manifest at the level of global gene expression regulation. The interaction between tumor promoting pathways and the DDR has implications in the tumor microenvironment; therefore, uncovering a gene signature associated with this synergistic interaction would be informative as a potential biomarker.

In this study, TK6 cells were pretreated with TPA followed by exposure to UVC-irradiation in order to determine the global transcriptional profile of TPA+UVC treated cells compared to that induced by either stress alone. We conducted two RNA-seq experiments to determine the time and dose dependent synergistic effects of the co-treatment. In this manner, we were able to systematically filter the differentially expressed genes and determine the synergistically altered pathways/genes. The resulting genes discovered with this approach were validated by treating TK6 cells with other PKC-activating tumor promoters and analyzing the expression by QPCR. The data presented here show how tumor promoter-induced signaling perturbation converge with DDR pathways triggered by UVC-irradiation. Identification of these key pathway nodes is important for elucidating the synergistic interactions that may underlie the mechanisms of tumor promotion.

## Materials and Methods

### Materials

TPA (CAS 16561-29-8, Sigma-Aldrich, St. Louis, MO), 4α-phorbol 12-myristate 13-acetate (4α-TPA) (CAS 63597-44-4, Sigma-Aldrich), phorbol-12,13-dibutyrate (PDBu) (CAS 37558-16-0, Sigma-Aldrich), sapintoxin D (CAS 80998-07-8, Sigma-Aldrich), mezerein (CAS 34807-41-5, Santa Cruz Biotechnology, Dallas, Texas), (-)-Indolactam V ((-)-Ind V) (CAS 90365-57-4, Santa Cruz Biotechnology) and resiniferonol 9,13,14-ortho-phenylacetate (ROPA) (CAS 57852-42-3, Santa Cruz Biotechnology) were diluted in sterile Hybri-Max™ dimethyl sulfoxide (DMSO) (CAS 67-68-5, Sigma-Aldrich) to 1000X concentrations and stored frozen.

### Experimental Design

The human lymphoblastoid TK6 cell line was purchased from the American Type Culture Collection (ATCC, CRL-8015, Manassa, VA) and propagated in RPMI medium (Mediatech, Manassas, VA) supplemented with 10% heat-inactivated fetal bovine serum (Mediatech) and penicillin/streptomycin (Mediatech). TK6 cells were pretreated with tumor promoters or a solvent control (DMSO, 0.1%) for 72 hours prior to UVC-irradiation in order to maximize the pathway deregulating effects of sustained exposure on the cells. Detailed descriptions of the tumor promoter pretreatment and the UVC-irradiation procedures have been described previously [13]. The cells were then maintained in media supplemented with the tumor promoting agent or DMSO for the remainder of the experiment. DMSO-treated cells were used as the untreated control in each experiment for differential expression analysis. In the time-course experiment, RNA was isolated at 4, 8 and 24 hours following UVC-irradiation or mock-irradiation conditions. The resulting treatment conditions were TPA+UVC, UVC-alone, TPA-alone and the vehicle treated control. The concentration of TPA was 1 nM and the cells were exposed to 10 J/m^2^ UVC. In the dose-response experiment TK6 cells were pretreated with three concentrations of TPA (0.2, 0.5 and 1 nm) or two concentrations of 4αTPA (1 and 10 nM) prior to UVC-irradiation (10 J/m^2^) and RNA was isolated after 8 hours. In each experiment, three biological replicates were used for RNA-seq for each treatment condition and time-point.

### RNA Isolation and Quality Control

At predefined time-points after UVC-irradiation the cells were removed from the incubator and washed twice with cold Dulbecco’s phosphate buffered saline (DPBS). The cells were then flash frozen on dry ice and stored at -80°C until further processing. RNA was isolated using an RNeasy kit (Qiagen, Valencia, CA) and potential DNA contamination was removed with DNase treatment (TURBO DNA-free™ Kit, Life Technologies). RNA concentration and quality was verified on a Nanodrop spectrophotometer (NanoDrop, Wilmington, DE). RNA integrity was determined with an Agilent Bioanalyzer 2100 (Agilent, Santa Clara, CA). All RNA samples sent for RNA-sequencing had a 260/280 absorption ratio that was greater than 2 and a RNA integrity number (RIN) that was greater than 9.8.

### RNA-Sequencing (RNA-Seq)

Total RNA was sent to the DuPont Pioneer Genomics Core Facility (Johnston, Iowa) for RNA-seq. RNA samples were prepared for sequencing with the Illumina TruSeq RNA preparation kit to purify for polyA RNA, fragment the samples and prime for cDNA synthesis. Samples were sequenced on an Illumina HiSeq 2000 using a standard single read protocol (50 bp read length). Images from the sequencing run were analyzed with the Illumina CASAVA pipeline (v1.8). The resulting sequences were filtered for bases with Q scores greater than 20 and trimmed accordingly. Sequences of less than 24 bp were removed. Filtered reads were aligned to consensus CDS (CCDS) sequences (GRCh37.p5, 20110907) with bowtie and the expression level was normalized by gene size by RPKM (reads mapping to CCDS per kilobase of transcript per million reads sequenced).

In the time-course experiment, 887 million reads (10–40 million/sample) were sequenced from 42 samples. Approximately 55% of the reads aligned to the reference genome of which 15% were aligned to multiple regions. Multiple aligned reads were removed leaving approximately 40% of the reads in the final analysis. No reads were mapped from one of the 4-hr TPA+UVC replicates (potential multiplexing error) and further analysis was conducted with duplicate instead of triplicate samples for this condition. In the dose-response experiment, 305 million reads (~10–25 million/sample) were sequenced from 21 samples. Approximately 50% of the reads aligned to the reference genome of which 12% were aligned to multiple regions. Multiple aligned reads were removed leaving approximately 38% of the reads in the final analysis.

In order to facilitate proper data processing for the downstream analysis, zero values were set to “NA” (not applicable) in instances where only one of the three replicates were missing reads for a specific gene. Differentially expressed genes were determined using analysis of variance (ANOVA) with a false discovery rate (FDR, Benjamini-Hochberg) of ≤0.05 and fold-change ±2 in Array Suite software. The data discussed in this publication have been deposited in NCBI's Gene Expression Omnibus [14] and are accessible through GEO Series accession number GSE71521 (http://www.ncbi.nlm.nih.gov/geo/query/acc.cgi?acc=GSE71521).

### Gene Set and Pathway Analysis

**Fig 1:**
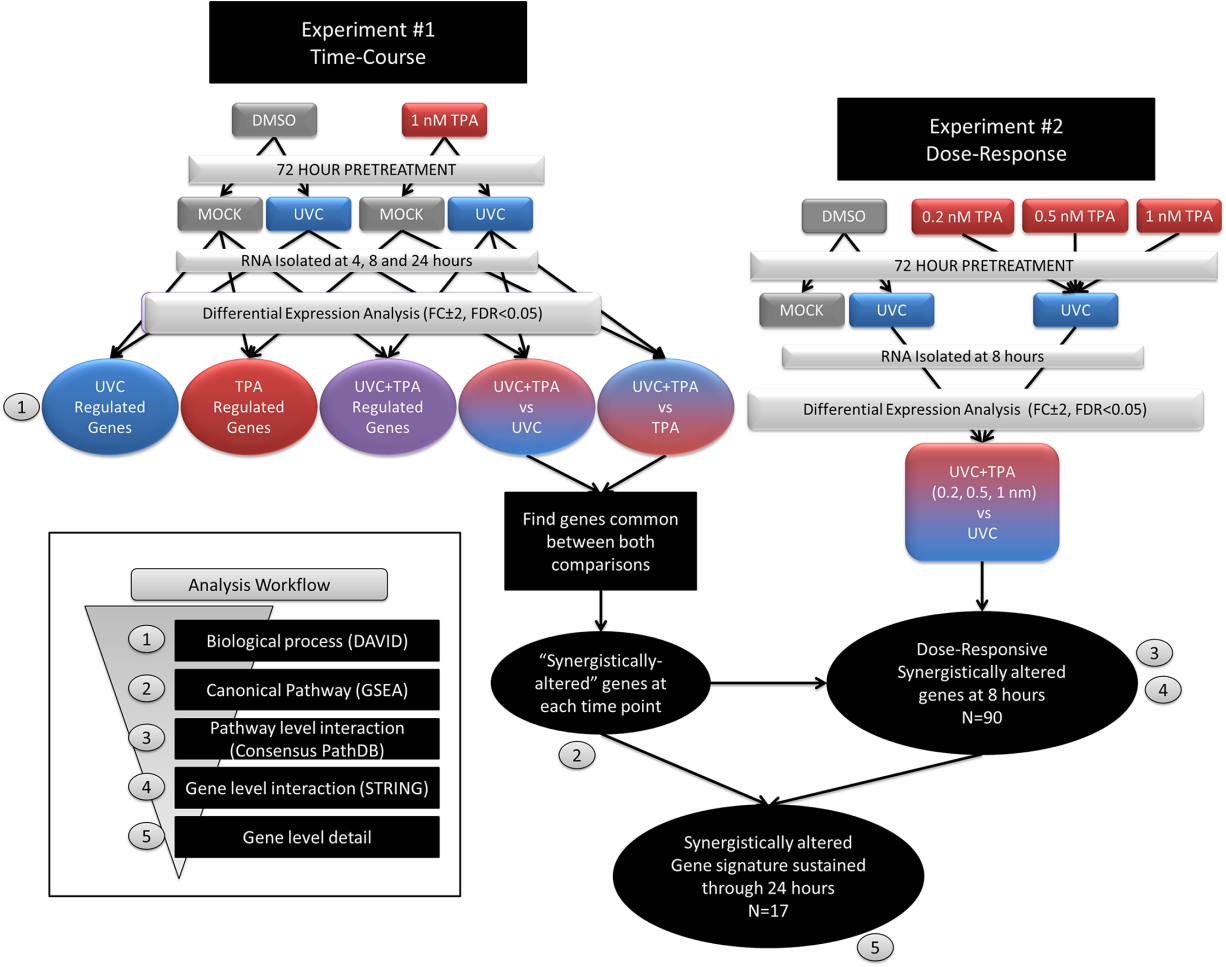
Gene set analysis workflow. Two separate experiments were conducted to determine the time- (experiment #1) and dose-responsive (experiment #2) gene expression patterns in the TPA+UVC co-treated cells. We filtered the gene set through an analysis workflow in order to uncover a synergistic, dose-responsive and reproducible gene signature associated with TPA+UVC exposure. Using several open-source analysis tools, we were able to describe different levels of biological complexity in the data. (1) First, differential expression analysis of TPA, UVC and the TPA+UVC treated cells versus the untreated control was analyzed for significant up or down regulation of biological processes (DAVID) at 4, 8 and 24 hours. Then, differential expression was determined for the TPA+UVC co-treated cells using TPA-alone or UVC-alone treated cells as the basis for comparison (i.e. the control samples). In this manner, we were able to determine the synergistically regulated genes in the TPA+UVC treated cells compared to either stress alone. (2) These synergistic genes were further analyzed to determine the canonical pathways significantly enhanced by TPA+UVC using gene set enrichment (MolSigDB). The synergistically regulated genes in experiment #1 (time-course) that were also dose-responsive in experiment #2 were analyzed for (3) pathway level (ConsensusPathDB) and (4) gene level interactions (STRING). (5) The genes found to be sustained (up to 24 hours) in the synergistic manner, in experiment #1, were considered the key gene signature that is specially enhanced by the combined treatment to TPA+UVC.

The workflow for gene set analysis is described in Fig 1 which describes how the biological context of the data analysis became increasingly focused as the gene sets were filtered. In this manner, we were able to capture large scale biological changes to pathway level perturbations and ultimately down to gene level interactions that could be linked to phenotypic endpoints based on expression patterns.

Differentially expressed genes (DEGs) were analyzed with several open-source pathway analysis databases. Functional annotation was conducted with DAVID version 6.7 to determine the significantly enriched biological processes represented in the gene lists (http://david.abcc.ncifcrf.gov/home.jsp) [15]. DAVID analysis was conducted under medium stringency for biological process enrichment (GOTERM_BP_FAT) and annotation clusters were summarized manually for representation purposes. To determine the overrepresentation of genes in canonical pathways, the TPA+UVC significantly altered gene lists were analyzed with the Molecular Signatures database version 4.0 (http://www.broadinstitute.org/gsea/msigdb/index.jsp) [16]. Overlap of genes represented in enriched pathways in the TPA+UVC gene signature was conducted with ConsensusPathDB Release 28 (23.01.2014) to determine a pathway level interaction network (http://cpdb.molgen.mpg.de) [17]. A protein level interaction network representing potential protein-protein interactions and sub-network clusters was determined with STRING version 9.1 (http://string-db.org/) with K-means clustering [18].

### Quantitative Real-Time PCR (QPCR)

TK6 cells were treated with additional PKC-activating tumor promoters as described previously for TPA. These compounds included PDBu (100 nM), Sapinotoxin D (100 nM), mezerein (100 nM), Ind-V (1000nM) and ROPA (1000 nM). The criteria for dose selection was described previously [13]. RNA was collected at 8-hours post UVC-irradiation. QPCR was run on an ABI 7500 Real-Time PCR system using ABI master mix and TaqMan® primers (Life Technologies). cDNA was generated with the SuperScript® VILO™ cDNA Synthesis Kit (Life Technologies) with a gradient thermocycler (Eppendorf, Hamburg, Germany). Gene expression levels were calculated using a standard curve for relative quantification with 3 biological replicates per sample. *18S rRNA* was used as the housekeeping gene for normalization purposes. Differential expression was determined by comparing against the vehicle treated, non-irradiated samples.

## Results

### TPA-Treated Cells Have a Synergistic Gene Expression Pattern in Response to UVC-Irradiation

**Fig 2:**
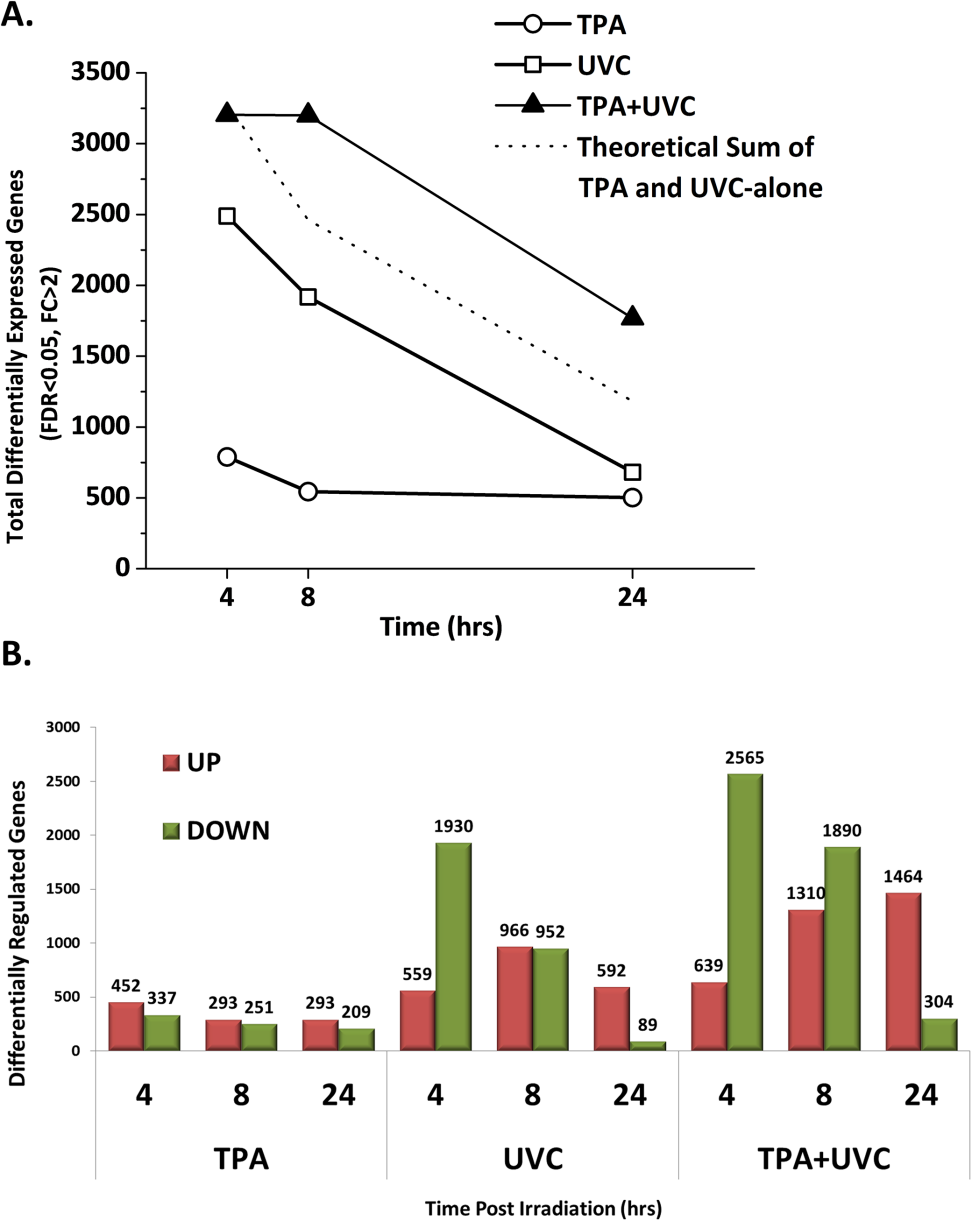
Synergistic gene expression in response to TPA+UVC treatment. (A) Compared to the non-irradiated vehicle control, there were more DEGs (fold-change ±2 and FDR<0.05) in the TPA+UVC treated cells compared to UVC or TPA alone. The total number of DEGs was higher than the theoretical sum of TPA and UVC alone and was sustained through 24 hours, representing a synergistic effect. (B) UVC caused mostly down-regulation early which was reversed by 24 hours. TPA+UVC caused a similar trend with a greater amount of DEGs at each time-point. TPA, in contrast, caused similar levels of up and down-regulation at each time point. Therefore, UVC treatment was more influential on overall gene expression patterns in the co-treated cells compared to TPA.

We analyzed the gene expression profile of TK6 cells exposed to PKC pathway dysregulation (TPA-treatment) and DNA damage (UVC-irradiation) to model synergistic effects that may result from activation of multiple stress response pathways in the tumor microenvironment. The gene expression profile of TPA+UVC co-treated cells was compared to that induced by TPA or UVC treatment alone at 4, 8 and 24 hours. Compared to UVC or TPA treatment alone, the TPA+UVC treated cells had significantly more differentially expressed genes (DEGs) at each time-point (Fig 2A). TPA+UVC treatment resulted in a total of 3204, 3200 and 1768 DEGs at 4, 8 and 24 hours respectively. By comparison, UVC treatment alone resulted in 2489, 1918 and 681 DEGs while TPA treatment alone resulted in 789, 544 and 502 DEGs at the same respective time-points. The total number of DEGs induced by the co-treatment exceeded the theoretical sum of the DEGs induced by either treatment alone at 8 and 24 hours, thus representing a synergistic response on the overall expression pattern.

Notably, a time-dependent trend in expression patterns was observed in the UVC-irradiated samples, which includes both the UVC-alone and TPA+UVC samples. In these samples, approximately 75–80% of the DEGs were down-regulated at 4 hours (Fig 2B). However, by 24 hours this trend was reversed and 80–85% of the DEGs were up-regulated instead. In the TPA-alone samples, no time dependent expression pattern was observed. Based on these findings, it becomes apparent that the total number and time-dependent expression pattern of the DEGs in the co-treated cells was more influenced by UVC-irradiation than the TPA-treatment.

Functional annotation (DAVID) of the DEGs revealed biological processes that were up or down- regulated by TPA, UVC or TPA+UVC treated cells compared to the untreated control at each time point (S1 and S2 Tables). The major biological processes altered by TPA treatment alone were associated with the inflammatory/immune response and were consistent across each time point. In contrast, the biological processes altered by UVC-irradiation alone were more time-dependent. At 4-hours, processes involved in phosphorylation/signal transduction, cell cycle, transcription, chromatin modification and cytoskeleton meditated processes were down-regulated by UVC-irradiation. At 8 hours, the p53 mediated DNA damage response was up-regulated and negative regulation of cell growth was down-regulated. By 24 hours, the top up-regulated processes were epithelial cell development and the inflammatory response. As expected, the top regulated biological processes induced by both TPA-alone and UVC-alone were often represented in the TPA+UVC gene sets as well. The most significant enriched pathways by TPA+UVC treatment, based on EASE score, were down-regulation of protein catabolic processes and cell cycle at 4 hours and up-regulation of inflammatory genes at each time-point which was most significant at 24 hours.

In our previous study, we characterized a synergistic increase in apoptosis that occurs in cells co-treated with TPA and UVC [13]. Therefore, in this experiment we analyzed the number of genes enriched for regulation of programmed cell death (GO:0043067) by TPA-alone, UVC-alone and TPA+UVC at each time-point (S3 Table). We observed significantly more GO:0043067 genes differentially expressed in the TPA+UVC condition compared to either stress alone at each time point. Specifically, there were 174, 191 and 129 apoptosis genes in the TPA+UVC condition compared to 135, 120 and 54 in the UVC-alone and 54, 48 and 40 in the TPA-alone at 4, 8 and 24 hours respectively. At both 8 and 24 hours, the number of apoptosis genes in the TPA+UVC condition was greater than the sum of either stress alone, indicating a synergistic effect.

### Synergistically Altered Genes Induced by TPA+UVC Treatment Compared to Either Stress Alone

To determine the DEGs specifically responsive to the combination of TPA and UVC treatment, we looked for genes significantly increased or decreased (fold change ±2 and FDR<0.05) by the TPA+UVC co-treatment when using UVC or TPA treated samples as the control at each time-point. TPA+UVC co-treatment resulted in 1746, 1862 and 834 DEGs compared to TPA-alone at 4, 8 and 24 hours respectively. Compared to UVC-alone, TPA+UVC co-treatment resulted in 244, 469 and 628 DEGs at 4, 8 and 24 hours respectively.

**Fig 3:**
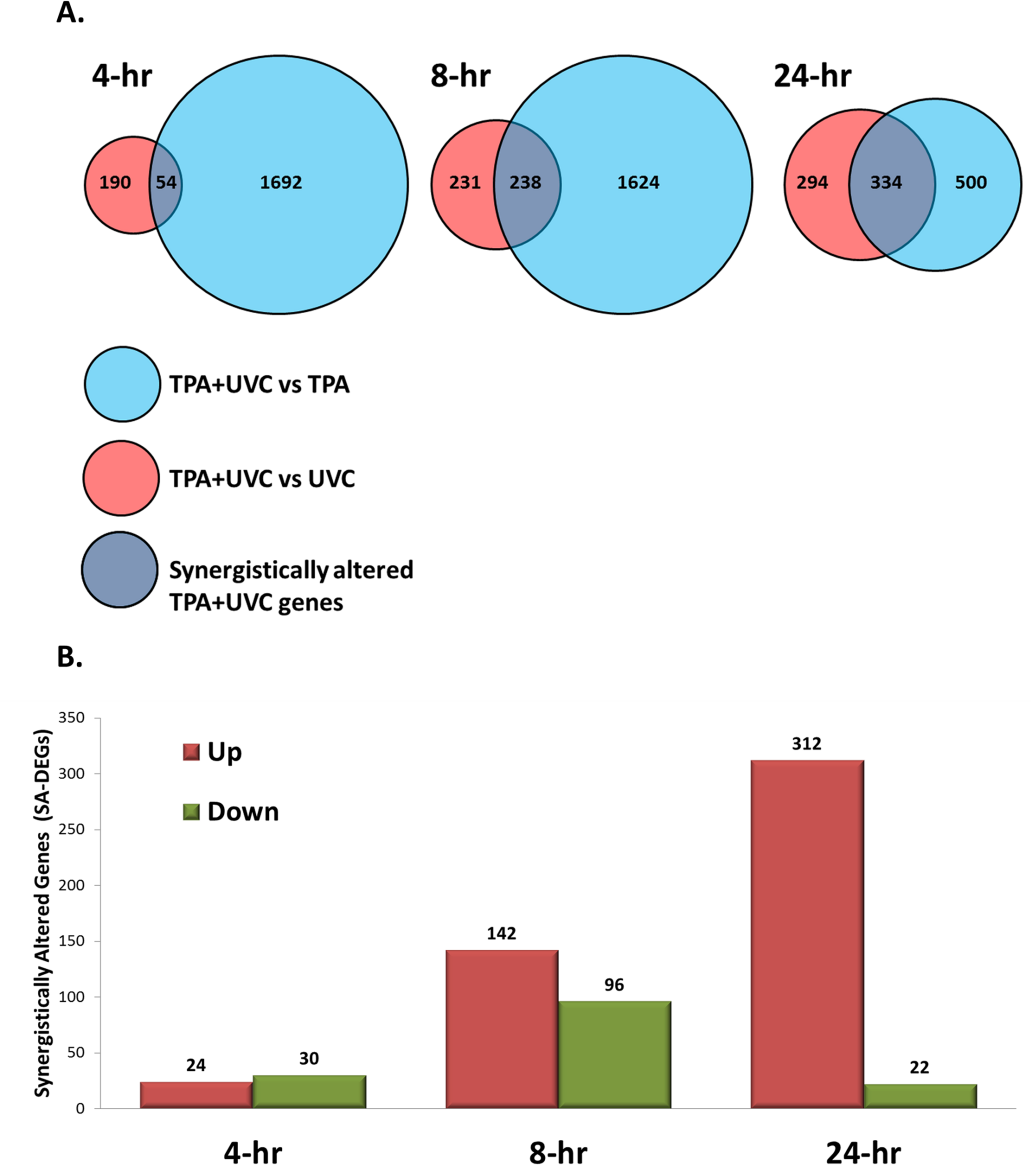
Determination of genes significantly altered in TPA+UVC treated samples compared to either stress alone. (A) Significantly altered DEGs (SA-DEGs) were considered genes differentially expressed (Fold change ±2 and FDR <0.05) in the TPA+UVC samples compared to both UVC and TPA treatment alone. (B) Despite large numbers of DEGs at the 4-hour time point, few of the genes were considered to be SA-DEGs in the TPA+UVC treated cells. Most of the SA-DEGs were observed in the 8 and 24-hour time points, with mostly synergistic up-regulation of expression observed in the latter.

We then determined the DEGs common between the result from the TPA+UVC vs TPA comparison and the TPA+UVC vs UVC comparison (Fig 3A). These genes were referred to as synergistically altered genes (SA-DEGs) because they are greater than 2 fold enhanced by the combined treatment when compared to either stress alone. We found 54, 238 and 334 SA-DEGs in the TPA+UVC treated cells at 4, 8 and 24 hours, respectively (Fig 3A). At 4 and 8 hours there was a similar distribution between significantly up- and down-regulated genes while at 24 hours most of the SA-DEGs were significantly up-regulated (312 up versus only 22 down) (Fig 3B). At each time point, a majority (>65%) of the SA-DEGs genes were categorized as unique genes (found differentially expressed only in the combined treatment but not by either UVC or TPA alone when compared to the untreated control, data not shown). Therefore, a significant portion of genes regulated by TPA+UVC treatment would not have been observed in the transcriptional profile of either stress alone.

The SA-DEGs at each time point were analyzed with the molecular signatures database [16] to determine the significantly perturbed canonical pathways at each time point (Table 1). At 4 hours, the 54 SA-DEGs were not statistically associated with any pathways listed in the database. At 8 hours, the 238 SA-DEGs were enriched for the AP1 pathway, GPCR ligand binding/signaling and the p53 downstream pathway. In addition, cytokine receptor interaction and interferon gamma signaling was also enriched at 8-hours indicating significantly perturbed inflammatory response genes as well. By 24 hours, the 334 SA-DEGs were strongly enriched for immune/cytokine/interferon/inflammatory response pathways.

**Table 1:** Canonical pathways synergistically altered by TPA+UVC co-treatment.

	Gene Set Name	# Genesin Gene Set (K)	Description	# Genes in Overlap (k)	k/K	FDR q-value
8-hr	PID_AP1_PATHWAY	70	AP-1 transcription factor network	6	0.0857	<0.001
	REACTOME_GPCR_LIGAND_BINDING	408	Genes involved in GPCR ligand binding	11	0.027	<0.01
	PID_P53DOWNSTREAMPATHWAY	137	Direct p53 effectors	7	0.0511	<0.01
	KEGG_CYTOKINE_CYTOKINE_RECEPTOR_INTERACTION	267	Cytokine-cytokine receptor interaction	9	0.0337	<0.01
	REACTOME_SIGNALING_BY_GPCR	920	Genes involved in Signaling by GPCR	16	0.0174	<0.01
	REACTOME_CLASS_A1_RHODOPSIN_LIKE_RECEPTORS	305	Genes involved in Class A/1 (Rhodopsin-like receptors)	9	0.0295	<0.01
	REACTOME_GPCR_DOWNSTREAM_SIGNALING	805	Genes involved in GPCR downstream signaling	13	0.0161	<0.05
	PID_ATF2_PATHWAY	59	ATF-2 transcription factor network	4	0.0678	<0.05
	KEGG_COLORECTAL_CANCER	62	Colorectal cancer	4	0.0645	<0.05
	REACTOME_INTERFERON_GAMMA_SIGNALING	63	Genes involved in Interferon gamma signaling	4	0.0635	<0.05
24-hr	REACTOME_IMMUNE_SYSTEM	933	Genes involved in Immune System	44	0.0472	<0.001
	KEGG_CYTOKINE_CYTOKINE_RECEPTOR_INTERACTION	267	Cytokine-cytokine receptor interaction	21	0.0787	<0.001
	REACTOME_CYTOKINE_SIGNALING_IN_IMMUNE_SYSTEM	270	Genes involved in Cytokine Signaling in Immune system	27	0.1	<0.001
	REACTOME_INTERFERON_SIGNALING	159	Genes involved in Interferon Signaling	20	0.1258	<0.001
	REACTOME_INTERFERON_ALPHA_BETA_SIGNALING	64	Genes involved in Interferon alpha/beta signaling	15	0.2344	<0.001
	KEGG_JAK_STAT_SIGNALING_PATHWAY	155	Jak-STAT signaling pathway	13	0.0839	<0.001
	PID_P53DOWNSTREAMPATHWAY	137	Direct p53 effectors	11	0.0803	<0.001
	REACTOME_INNATE_IMMUNE_SYSTEM	279	Genes involved in Innate Immune System	14	0.0502	<0.001
	PID_ATF2_PATHWAY	59	ATF-2 transcription factor network	8	0.1356	<0.001
	REACTOME_ANTIVIRAL_MECHANISM_BY_IFN_STIMULATED_GENES	66	Genes involved in Antiviral mechanism by IFN-stimulated genes	8	0.1212	<0.001

### Dose-Dependent Gene Expression Pattern of TPA-Treated Cells Exposed to UVC-Irradiation

In our previous study, we observed a TPA dose-dependent increase in the sensitivity to UVC-irradiation in TK6 cells characterized by a synergistic increase in apoptosis and delayed DNA repair observed as sustained γH2AX signaling [13]. In addition, we also observed altered DNA damage induced gene expression in TPA-pretreated cells at both 8 and 24 hours post UVC-irradiation [13]. Therefore, we used a similar dose-range in this study to determine the synergistic gene expression patterns at 8 hours post UVC-irradiation. We determined the dose-responsive DEGs induced by low (0.2 nM), mid (0.5 nM) and high (1.0 nM) TPA treatment combined with UVC-irradiation and compared the expression profile with that induced by UVC-alone. In addition, we also included two concentrations of the non-tumor promoting phorbol ester 4α-TPA, as an additional control (4α-TPA is an inactive isomer of TPA). To determine the SA-DEGs that occurred in a dose-dependent trend, we looked for genes that were differentially expression in the TPA+UVC samples compared to UVC-alone that followed an increasing (0.2 nM TPA+UVC ≤ 0.5 nM TPA+UVC ≤ 1.0 nM TPA+UVC) or decreasing (0.2 nM TPA+UVC ≥ 0.5 nM TPA+UVC ≥ 1.0 nM TPA+UVC) dose-responsive pattern. In addition, the SA-DEGs were also differentially regulated (Fold change ± 2, FDR <0.05) in 1.0 nm TPA+UVC treated cells compared to the non-irradiated, vehicle control. We found 679 genes that followed this trend (332 were dose-dependent up-regulated and 347 were dose-dependent down-regulated). In contrast, the non-tumor promoter, 4α-TPA+UVC treated cells only had 66 or 85 DEGs (Fold change ± 2, FDR <0.05) in the co-treated samples compared to UVC-alone at 1 and 10 nM, respectively (S1 Fig).

**Fig 4:**
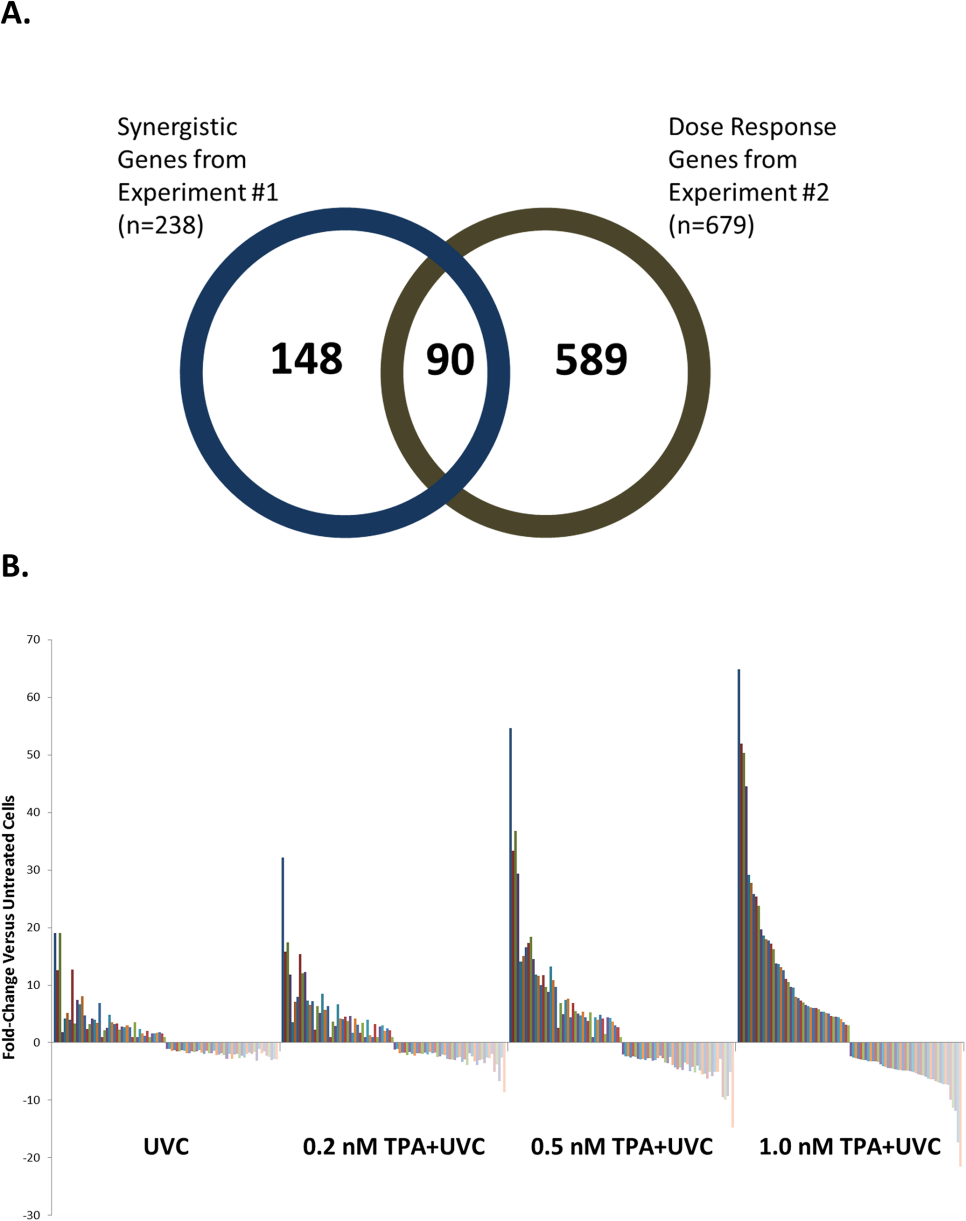
TPA+UVC 8-hour gene signature. (A) A 90 gene signature was derived by determining genes that were significantly altered compared to either stress alone in the time-course experiment (experiment #1) at 8-hours and also expressed in a TPA dose-responsive trend in experiment #2. (B) Visualization of the 90 gene signature based on fold-change in the dose-response experiment revealed the increasing or decreasing expression trends for each gene (each series represented by individual gene in signature) as the concentration of TPA increased from 0.2 nM to 1.0 nM.

We then further filtered the dose-responsive gene list by comparing it to the 238 SA-DEGs found in the time-course experiment at the 8-hour time point (Fig 4A). The 90 overlapping genes between both gene sets represented only genes that are both dose-dependently regulated and occur in a synergistic response to the co-treatment of TPA and UVC. (Fig 4B).

**Fig 5:**
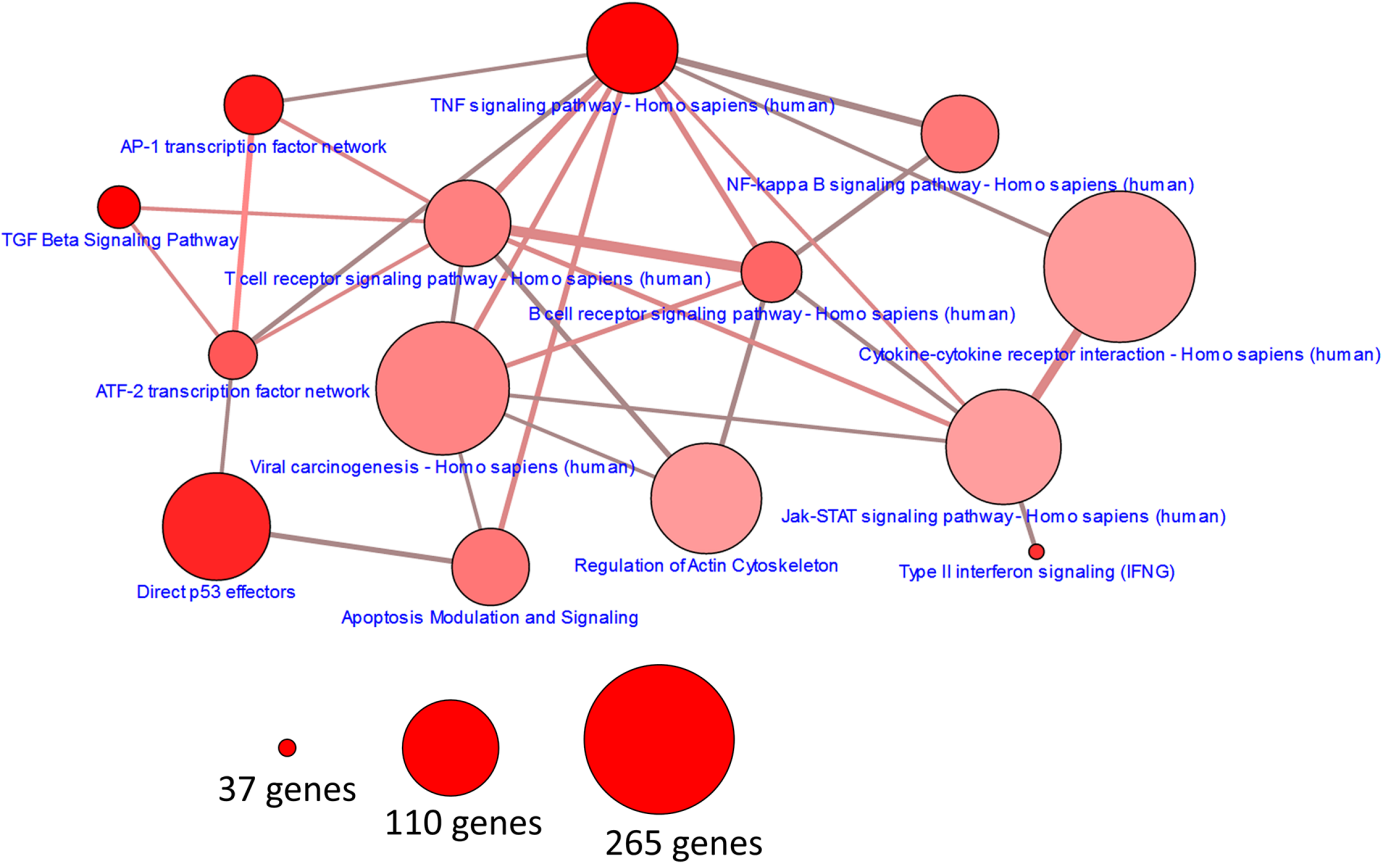
Pathway enrichment of TPA+UVC gene signature. Enrichment of signaling and inflammatory pathways connected by at least one gene in the TPA+UVC gene signature is represented (ConsensusPathDB). The node size is based on number of genes represented in each pathway gene set. The color is determined by statistical enrichment value (darker red = lower p value). The edge thickness represents the total number of genes shared between each pathway with the edge color representing the number of genes in the gene signature shared between each pathway (darker red = more genes). The most significantly enriched pathways were TNFα, TGFβ, IFNγ, p53 and AP-1.

The resulting 8-hour gene signature was analyzed using the consensus path database to determine the significantly enriched pathways and the genes found in multiple pathways [17] (Fig 5). The top enriched pathways were those associated with the inflammatory response such as TGFβ, TNFα and interferon in addition to p53 and AP1. When represented in the consensus path database, many of the enriched pathways have at least a 10% overlap of genes and at least one gene in the TPA+UVC gene signature shared between them (edges in represented pathway network). The individual genes associated with each pathway are listed in Table 2.

**Table 2:** Overlap of the signaling pathways enriched in the dose-dependent and synergistic genes induced by TPA+UVC co-treatment.

Pathway name	set size	candidates	p-value	q-value	pathway source	genes
TGF Beta Signaling Pathway	55	4 (7.3%)	7.92E-05	0.00165	Wikipathways	*SERPINE1*, *IFNG*, *LIF*, *FOS*
TNF signaling pathway—Homo sapiens (human)	110	5 (4.5%)	9.17E-05	0.00165	KEGG	*PIK3CD*, *TRAF5*, *MAP3K5*, *FOS*, *LIF*
AP-1 transcription factor network	70	4 (5.7%)	0.000204	0.00275	PID	*FOSB*, *FOS*, *ATF3*, *IFNG*
Direct p53 effectors	142	5 (3.5%)	0.000304	0.00328	PID	*LIF*, *ATF3*, *SERPINE1*, *GDF15*, *PMAIP1*
Type II interferon signaling (IFNG)	37	3 (8.1%)	0.000487	0.00439	Wikipathways	*CIITA*, *GBP1*, *IFNG*
ATF-2 transcription factor network	60	3 (5.0%)	0.00201	0.00972	PID	*ATF3*, *FOS*, *IFNG*
B cell receptor signaling pathway—Homo sapiens (human)	72	3 (4.2%)	0.00338	0.014	KEGG	*PIK3CD*, *FOS*, *LYN*
Apoptosis Modulation and Signaling	90	3 (3.3%)	0.00631	0.0207	Wikipathways	*MAPK35*, *FOS*, *PMAIP1*
NF-kappa B signaling pathway—Homo sapiens (human)	91	3 (3.3%)	0.00651	0.0207	KEGG	*TRAF5*, *CD14*, *LYN*
T cell receptor signaling pathway—Homo sapiens (human)	104	3 (2.9%)	0.00939	0.0241	KEGG	*PIK3CD*, *FOS*, *IFNG*
Viral carcinogenesis—Homo sapiens (human)	206	4 (1.9%)	0.0107	0.0241	KEGG	*TRAF5*, *PIK3CD*, *PMAIP1*, *LYN*
Regulation of Actin Cytoskeleton	148	3 (2.0%)	0.024	0.046	Wikipathways	*CD14*, *PIK3CD*, *SSH3*
Cytokine-cytokine receptor interaction—Homo sapiens (human)	265	4 (1.5%)	0.0247	0.046	KEGG	*TNFSF4*, *BMPR1A*, *IFNG*, *LIF*
Jak-STAT signaling pathway—Homo sapiens (human)	156	3 (1.9%)	0.0275	0.0496	KEGG	*PIK3CD*, *IFNG*, *LIF*

**Fig 6:**
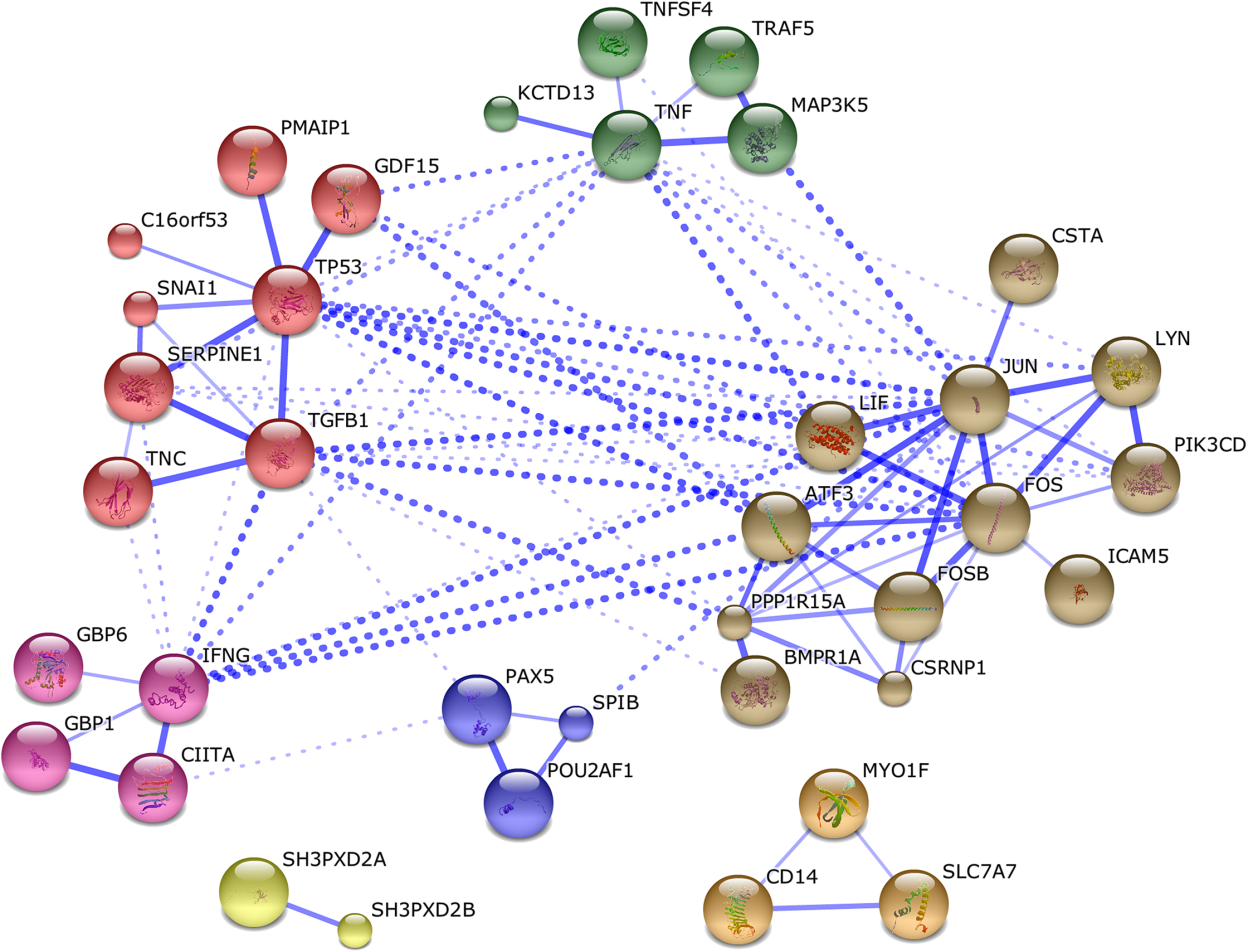
Network analysis of TPA+UVC gene signature. STRING analysis revealed key sub-network clusters and potential gene level interactions connected to *p53*, *TGFβ*, *TNF and JUN*. Colors represent different subnetworks based on K-means clustering. Edge thickness is representative of confidence in interaction based on database mining, experimental evidence and text mining.

The gene signature was further explored to determine protein interactions using the STRING database [18]. To determine the connections between key pathway nodes revealed previously, we imported p53, TGFβ1, TNF and JUN (co-factor in AP1 transcription factor) into the analysis. STRING analysis revealed 5 interconnected sub-networks in the gene signature including an AP1 cluster (11 genes), a p53 and TGFβ cluster (6 genes), a TNFα cluster (4 genes), an IFNγ cluster (4 genes) and a B-cell transcription factor cluster (3 genes) (Fig 6). Two other sub-networks were also found but not connected to the larger network of protein interactions including a SH3 domain cluster based on protein homology (2 genes) and an uncharacterized cluster with CD14 based on co-expression.

### Sustained Gene Signature Specific to TPA+UVC Co-Treatment

**Fig 7:**
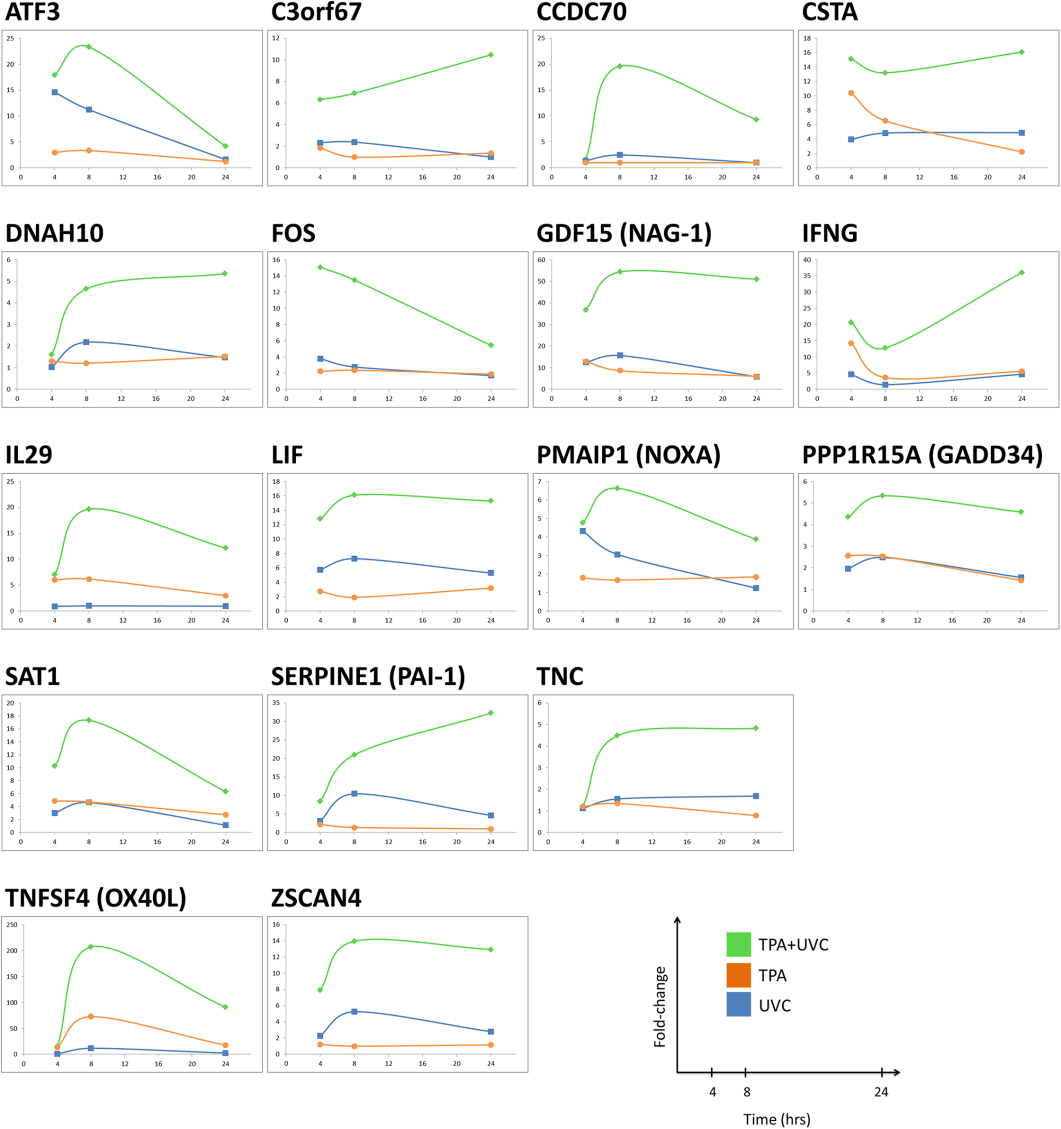
Sustained seventeen gene signature through 24 hours. Seventeen genes in TPA+UVC gene signature were significantly altered (fold-change ±2 and FDR <0.05) compared to UVC or TPA alone at both 8 and 24 hours.

**Fig 8:**
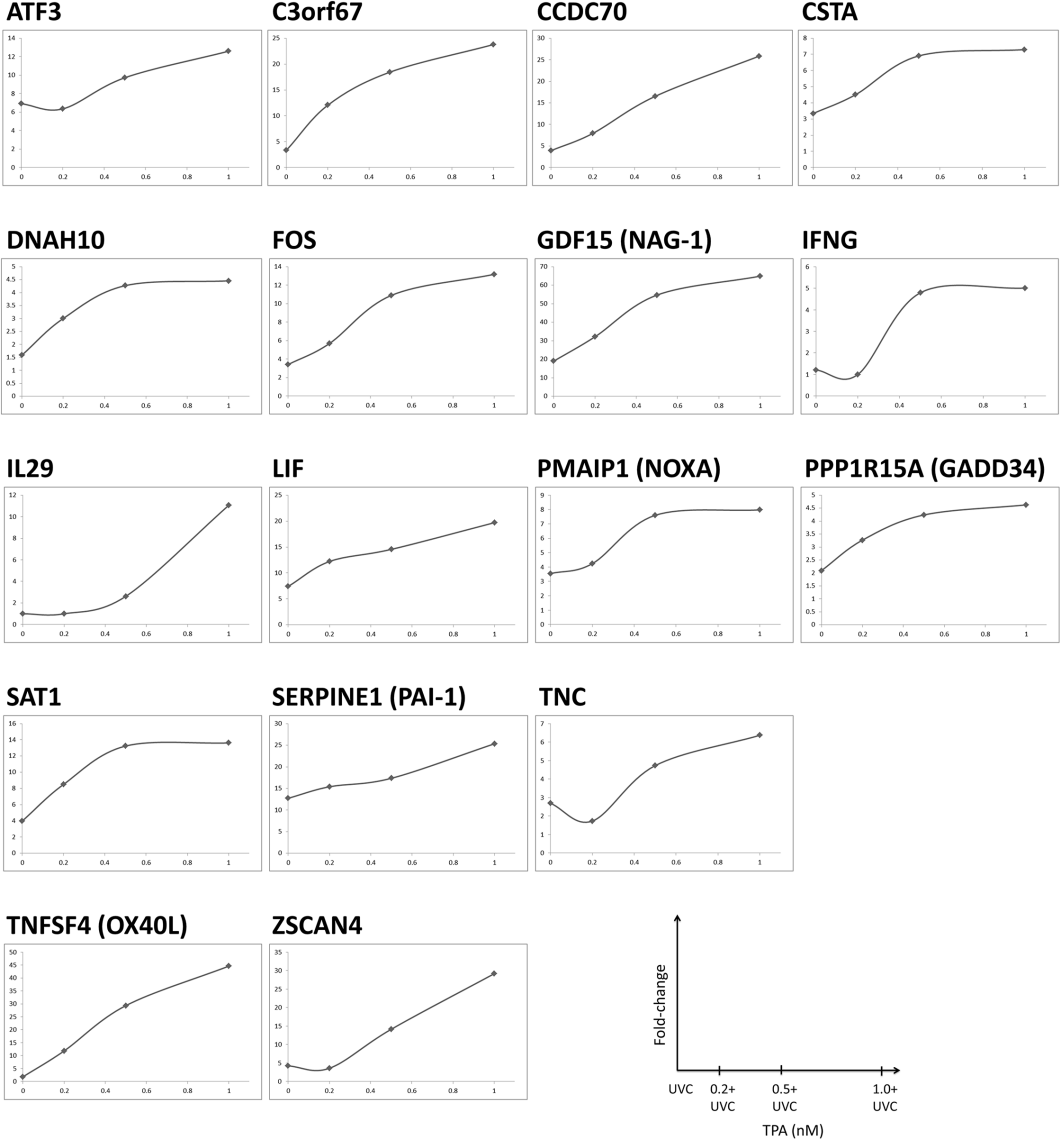
Dose-dependent expression of seventeen gene signature. The seventeen gene signature was TPA dose-responsive in co-treated cells at 8 hours post-UVC irradiation.

As a next step, we looked for genes in the 8 hour TPA+UVC gene signature that remained significantly altered compared to either stress alone at 24 hours. In this regard, these genes represented the delayed recovery and sustained DDR activation that occurs in UVC-irradiated TK6 cells treated with TPA [13]. For instance, TPA+UVC treatment caused sustained γH2AX signaling beginning 8 hours after UVC-irradiation that was maintained through 24 hours resulting in a synergistic increase in apoptosis [13]. In this study, seventeen of the 90 TPA+UVC SA-DEGs in the 8-hour gene signature remained significantly perturbed at 24 hours. These genes were synergistically enhanced in the TPA+UVC treated cells compared to either stress alone at both 8 and 24 hours (Fig 7) and occur in a dose-responsive trend at the 8-hour time-point (Fig 8). Most of the 17 genes were also found in the consensus path database pathway interaction network, which are *ATF3*, *CSTA*, *FOS*, *GDF15*, *IFNG*, *LIF*, *PMAIP1*, *PPP1R15A*, *SERPINE 1*, *TNC* and *TNFSF4*. The genes not enriched in the consensus path db network were *C3orf67*, *CCDC70*, *DNAH10*, *IL29*, *SAT1* and *ZSCAN*. Based on the filtering criteria used in this experiment, it was concluded that these 17 genes best represent the phenotypic responses described in our previous study based on the dose-dependent and sustained expression patterns. Detailed information of each of the 17 genes is described in Table 3.

**Table 3:** Phenotypically anchored TPA+UVC genes based on synergistic expression, dose-dependency and sustained activation.

Gene	Gene ID	Gene Name (ALIAS)	Function
ATF3	467	activating transcription factor 3	Transcription factor that binds to cAMP response element (CRE)
C3orf67	200844	chromosome 3 open reading frame 67	Unknown function
CCDC70	83446	coiled-coil domain containing 70	Secreted signaling protein
CSTA	1475	cystatin A (stefin A)	Thiol proteinase inhibitor involved in cell adhesion
DNAH10	196385	dynein, axonemal, heavy chain 10	Force generating protein with ATPase activity involved in cellular migration
FOS	2353	FBJ murine osteosarcoma viral oncogene homolog	Subunit of AP-1 transcription factor; forms heterdimers with JUN to activate transcription
GDF15	9518	growth differentiation factor 15 (NAG-1)	Secreted cytokine; part of TGFβ superfamily
IFNG	3458	interferon, gamma	Secreted cytokine; type II interferon
IL29	282618	interferon, lambda 1	Secreted cytokine; ligand for the heterodimeric class II cytokine receptor
LIF	3976	leukemia inhibitory factor	Secreted cytokine; involved in differentiation
PMAIP1	5366	phorbol-12-myristate-13-acetate-induced protein 1 (NOXA)	Propapoptotic protein; stimulates mitochondrial membrane depolarization and caspase activation
PPP1R15A	23645	protein phosphatase 1, regulatory subunit 15A (GADD34)	Stress response protein involved in recovery and apoptosis
SAT1	6303	spermidine/spermine N1-acetyltransferase 1	Enzyme that catalyzes the acetylation of polyamines
SERPINE1	5054	serpin peptidase inhibitor, clade E (PAI-1), member 1	Secreted protease inhibitor; binds to tissue plasminogen activator to inhibit activity
TNC	3371	tenascin C	Secreted extracellular matrix protein
TNFSF4	7292	tumor necrosis factor (ligand) superfamily, member 4 (OX40L)	Secreted cytokine; stimulates T-lymphocyte activation
ZSCAN4	201516	zinc finger and SCAN domain containing 4	Transcription factor that regulates stem cell pluripotency; regulates telomere elongation

### Validation of the Sustained Gene Signature with Other PKC-Activating Tumor Promoters

**Fig 9:**
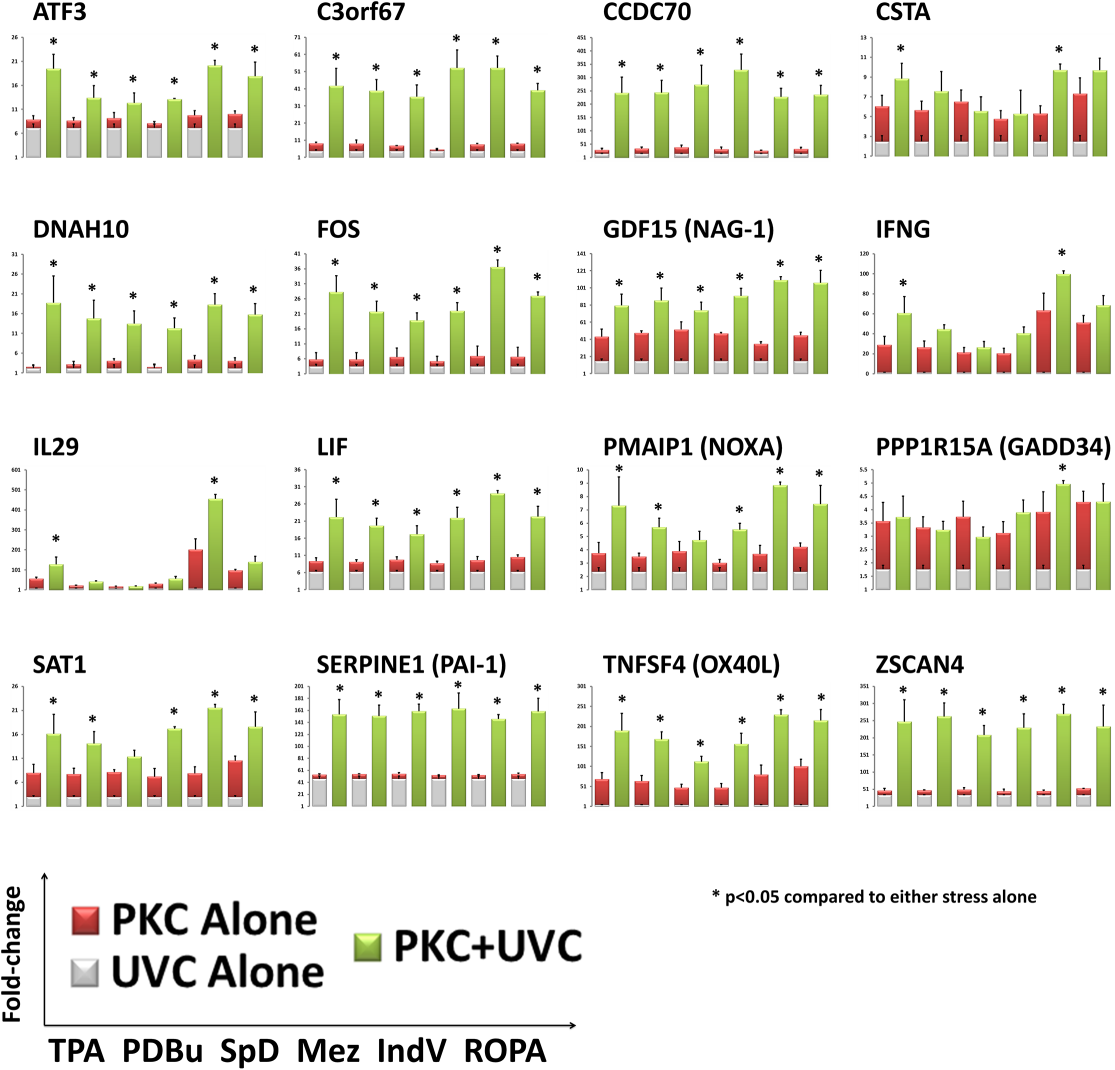
Validation of seventeen gene signature with other PKC-activating tumor promoters. TK6 cells were treated with other PKC-activating tumor promoters including PDBu, SPd, Mez, IndV and ROPA and exposed to UVC-irradiation to verify the 17 gene signature observed with RNA-sequencing. Gene expression was observed with QPCR. Most of the 17 genes had synergistic expression patterns in the co-treated cells (green bar). The fold-change of the UVC-alone (grey bar) and PKC-activator alone (red bar) are stacked to represent the theoretical additive response. One way ANOVA with Bonferroni post-hoc analysis was conducted (p<0.05) to determine statistical significance of the co-treated cells compared to either stress alone.

RNA was collected from UVC-irradiated TK6 cells pretreated with TPA, or other PKC-activating tumor promoters including PDBu, Sapinotoxin D, mezerein, Ind-V and ROPA. The resulting gene expression patterns were analyzed by QPCR. The other PKC-activating tumor promoting chemicals induced a similar synergistic gene expression pattern for *ATF3*, *C3orf67*, *CCDC70*, *DNAH10*, *FOS*, *GDF15*, *LIF*, *PMAIP1*, *SAT1*, *SERPINE1*, *TNFSF4 and ZSCAN4* (Fig 9). However, variable expression was observed for *CSTA*, *IFNG*, *IL29 and PPP1R15A* across the other compounds and synergistic effects were not readily observed despite an additive trend across the class. Additionally, differential expression of *TNC* could not be quantified by QPCR due to low level of expression and lack of amplification in approximately half of the samples analyzed. Based on RPKtM values in the RNA-seq data set, *TNC* had the lowest percentage of reads per CCDS sequence of the 17 gene signature (S4 Table). Therefore, it was assumed that QPCR was unable to amplify *TNC* in all of the samples due to Poisson distribution at low numbers (i.e. no template in some of the samples).

## Discussion

Carcinogenesis is a multistage process in which cells selectively become resistant to growth regulation and progressively develop advantageous mutations in oncogenes and tumor suppressors. This multistage process is influenced by a multitude of intra- and extracellular stressors that simultaneously activate stress response pathways and influence the selective outgrowth of cancer cells. Many of these stress response pathways can cross-talk leading to the convergence of multiple signal transduction events on similar kinases and transcription factors. It is anticipated that synergistic effects on gene expression likely occur in the tumor microenvironment due to the convergence of these stress response pathways.

In our previous study, we modeled how cells respond to multiple stressors by pretreating cells with different PKC-activating tumor promoters and challenging them with DNA damage via UVC-irradiation [13]. Tumor promoter pretreated cells had an exacerbated and sustained DNA damage response characterized by prolonged γH2AX formation and a synergistic increase in apoptosis after UVC-irradiation. In addition, co-treated cells had significantly perturbed expression of p53-target genes compared to cells exposed to UVC-alone. These phenotypic effects described previously were both time and TPA dose-dependent and therefore used to anchor gene expression patterns identified with RNA-seq in this study.

In this study, we used a systematic approach to filter the differentially expressed genes in order to uncover those that were synergistic in the combined treatment, TPA dose-dependent and repeatable between RNA-seq experiments. In this manner, we were able to determine a robust set of genes that fit these criteria and could be used to describe the interaction between two stress response pathways important in carcinogenesis.

The total number of differentially expressed genes in the TPA+UVC co-treated cells was greater than the sum of the differentially expressed genes by either stress alone at 8 and 24 hours, and therefore determined to be a synergistic response at these time points. However, at 4 hours the total number of differentially expressed genes in the co-treated cells was approximately equal to the theoretical additive response. Interestingly, this pattern was observed previously at the phenotypic level, as γH2AX was synergistically induced at 8 and 24 hours in TPA+UVC co-treated cells but at 4 hours the response was no greater than that induced by UVC-alone [13]. Therefore, while the total number of DEGs was highest at 4 hours when compared with 8 and 24 hours, the significance of this total is negligible when looking specifically for synergy between TPA and UVC induced stress at the gene expression level. This point was affirmed when we looked for genes that were significantly expressed (>2 fold-change, FDR<0.05) in the TPA+UVC treated cells compared to UVC or TPA treatment alone. Despite the high number of DEGs in the TPA+UVC treated cells (n = 3204 compared to the untreated control) at 4 hours, only 54 were considered to have synergistic expression. Interestingly the number of SA-DEGs in the TPA+UVC treated cells increased with time (n = 54 at 4 hours, n = 238 at 8 hours and n = 334 at 24 hours), while the total number of DEGs verses the untreated control was reduced with time (n = 3204 at 4 hours, n = 3200 at 8 hours and n = 1768 at 24 hours). Therefore, there was a noticeable temporal effect of the co-treatment when looking at synergistically expressed genes that otherwise would not have been observed.

To further refine the SA-DEGs, we conducted a second RNA-seq experiment to capture genes that were TPA dose-responsive at 8 hours. In this manner, we found a 90 gene signature that was synergistically expressed in the TPA+UVC treated cells, TPA-dose responsive, and repeatable between RNA-seq experiments. Pathway analysis revealed that this 90 gene signature represented the intersection of multiple signaling pathways including AP1, p53 and several inflammatory pathways. These pathways are well established targets of both TPA and UVC stress and therefore, this finding supported our hypothesis that the convergence of these stressors results in synergistic effects on gene regulation.

AP1 is a complex of individual subunits belonging to the c-Jun and c-Fos subfamilies that homo and heterodimerize and induce the expression of a multitude of immediate early response genes in response to growth factors, cytokines and stress [19]. In fact, many AP-1 transcriptional targets have TPA-response elements (TRE) in their promoters which are characterized as consensus binding sites for AP-1 [20]. Although a lot of work has focused on the oncogenic and transforming potential of AP-1 based on its activation by TPA and overexpression of h-Ras, many members of the AP-1 transcription factor family are also linked to tumor suppressors like p53 and are induced in response to DNA damage [21]. For instance, c-Jun and c-Fos are upregulated in response to DNA damaging agents such as UV, ionizing radiation (IR), hydrogen peroxide and DNA reactive chemicals [22–24]. Therefore, cells exposed to TPA stress and DNA damage simultaneously would be expected to have enhanced expression of genes linked to AP1 and p53. In this model, this effect was manifested as a synergistic response at the level of gene regulation.

In addition to AP1 and p53, the SA-DEGs were also highly associated with inflammatory pathways such as TGFβ, TNFα and IFNγ. This was particularly apparent at the 24 hour time point where many of the SA-DEGs were linked to the inflammatory response. These genes are likely representative of the high level of stress in co-treated cells that do not recover from the DNA damage. For example, the TNF superfamily death receptor activating protein *TRAIL* [25] was induced 7.6-fold compared to the untreated control in the TPA+UVC treated cells. In contrast, *TRAIL* was not induced in the cells exposed to TPA or UVC alone. Interestingly, many interferon inducible genes were also significantly induced by the TPA+UVC treatment (*IFIT1*, *IFIT2*, *ISG15*, *IFI6*, *IFIH1*, *IFI27*, *OAS1*, *IFI16*, *IFI44*, *IFNG*, *IFNB1*) which have also been shown to be associated with genotoxicity induced senescence in HeLa cells [26]. Overall, the significantly enhanced genes in the TPA+UVC treated cells at 24 hours were representative of the delayed recovery and increased cell death observed in TPA+UVC treated cells compared to UVC-irradiation or TPA-treatment alone which was reported previously [13].

To model this lack of recovery, we further filtered the 90 gene signature to determine which genes remained synergistically induced at 24 hours. Seventeen genes followed this trend, most of which were linked to the intersecting pathways described above. Many of these genes were also synergistically induced by other PKC-activating tumor promoters (including PDBu, Sapinotoxin D, mezerein, Ind-V and ROPA) in cells co-treated with UVC-irradiation. Therefore, these gene expression patterns were reproducible across chemical class, further confirming that these genes are specifically responsive simultaneous exposure to tumor promoting and DNA damaging stress.

Interestingly, many of these genes have been previously implicated in carcinogenesis. Despite the growth inhibitory functions of GDF15 and PAI-1, both are negatively correlated with cancer prognosis associated with multiple malignancies [27–29]. In fact, PAI-1 has been shown to be required for progression and metastasis in mice [30]. ATF3, a member of the AP-1 transcription factor family, has also been linked to carcinogenesis and is overexpressed in human tumors [31]. In addition to *GDF15*, *PAI-1 and ATF3*, we also observed many other genes that code for secreted proteins and cytokines including *IFNG*, *LIF*, *TNC*, *TNFSF4* and *IL29* were enhanced in the TPA+UVC gene signature and linked to TNFα, IFN-γ and/or TGFβ pathways indicating a strong paracrine signaling influence associated with the co-treatment. Because most of these genes subside by 24 hours in cells exposed to either stress alone, the enhanced expression of these genes may represent extracellular signaling triggered by a lack of recovery from damage in TPA+UVC treated cells. Previous studies have shown that ionizing radiation causes p53-dependent secretion of growth inhibitory factors which induces a bystander effect in non-irradiated cells [32]. In addition, p53-induced senescence has been linked with the release of extracellular matrix modifying proteins and inflammatory cytokines that contribute to aging and tumor promotion [33]. Considering that TPA and other PKC-activating compounds can induce cellular senescence in certain cancer lines, it is possible that exposure to both DNA damage and TPA simultaneously in this model results in exacerbated expression of senescence associated gene products involved in extracellular signaling and inflammation [34].

Inflammatory gene products have previously been shown to be induced in TK6 cells exposed to other genotoxic agents, particularly those that result in oxidative DNA damage [35–38]. Several of these genes were also induced upon TPA+UVC treatment in our study, including *GDF15*, *ATF3*, *FOS*, *NOXA*, *TNFSF4*, *IL29* and *IFNγ* [35]. Also, these genes appear to share similar upstream regulators that were described in this study, such as p53, TNF and TGFβ [36]. Therefore, TPA appears to synergistically enhance the expression of inflammatory genes in TK6 cells exposed to genotoxic agents which may be related to an increase in oxidative damage. However, we found no evidence on increased reactive oxygen species upon TPA+UVC co-treatment (S2 Fig). In addition, TPA induced a significantly different gene expression profile when compared to other genotoxic agents, including those that induce oxidative stress, in TK6 cells exposed for 4 hours [39]. Therefore, it is unlikely that oxidative DNA damage is the primary mechanism by which TPA enhances the expression profile of DNA damage inducible genes. In addition, several studies have shown a significantly different transcriptional profile induced by genotoxic carcinogens versus non-genotoxic carcinogens indicating different carcinogenic modes-of-action between these chemical classes [40–44]. The genes observed to be synergistically responsive to the TPA+UVC co-treatment in this experiment are hypothesized to be a result of disrupted upstream signal transduction pathways that occurs when TPA and DNA damage stress converge.

Lastly, we also observed several genes that may play a novel role in the intersection of tumor promoter and DNA damaging stress. These genes were C3orf67 (unknown function), CCDC70 (secreted signaling protein), DNAH10 (migration) and IL29 (secreted cytokine). Considering the strong synergistic expression compared to either stress alone, more research is warranted to determine the link between these genes and carcinogenesis.

In this study, we used a novel approach to determine what genes and pathways are synergistically induced in response to interacting stressors that are important in cancer. By looking at only the synergistically responsive genes, we could identify a subset of genes that could be anchored to phenotypic effects that otherwise would not have been observed. Because this study was conducted in an *in vitro* model, further validation is required *in vivo* to establish these synergistically responsive genes as critical factors in the process of multistage carcinogenesis.

## Supporting Information

S1 Fig Overlap of synergistically altered DEGs in the TPA+UVC and 4α-TPA+UVC samples compared to UVC-alone. Differential expression (Fold-change ±2, FDR<0.05) was determined in the co-treated cells using the UVC-alone treatment as the control. Therefore, these genes were considered synergistically altered genes. (A) The number of SA-DEGs increased with the dose of TPA. Many of the 0.2+TPA SA-DEGs are also represented in the 0.5 nM and 1.0 nM TPA+UVC gene sets. (B) Little overlap of SA-DEGs was observed between two concentrations of the non-tumor promoting phorbol ester 4α-TPA+UVC and the 1.0 nM TPA+UVC.
(TIF)

S2 Fig TPA did not increase ROS formation in TK6 cells with or without UVC-irradiation. TK6 cells were analyzed at 1 hour after UVC-irradiation for increased ROS formation with a live cell oxidative stress probe (CellROX® Green Reagent, Life Technologies) using flow cytometry. Ten-thousand cells were analyzed per condition for untreated cells (black line), UVC-alone (green), TPA-alone (red) and TPA+UVC (blue). tert-Butyl hydroperoxide (TBHP) (orange) was used as a positive control. TPA-pretreated cells appeared to have less ROS based on a slight population shift in probe fluorescence. Other time points were also analyzed including 2, 4 and 8 hours post-irradiation with similar findings as the 1 hour time-point (data not shown).
(TIF)

S1 Table Functional annotation summary of down-regulated genes by each treatment condition (DOCX)

S2 Table Functional annotation summary of up-regulated genes by each treatment condition (DOCX)

S3 Table Number of genes in each conditions associated with GO:0043067 Regulation of Programmed Cell Death. (DOCX)

S4 Table Log transformed RPKtM values for each of the 17 key genes. (DOCX)
