# Supplementary figures and images for: Synergistic Gene Expression Signature Observed in TK6 Cells upon Co-Exposure to UVC-Irradiation and Protein Kinase C-Activating Tumor Promoters
Source: PLoS One. 2015 Oct 2;10(10):e0139850. doi: 10.1371/journal.pone.0139850 (PMC4592187; doi:10.1371/journal.pone.0139850)

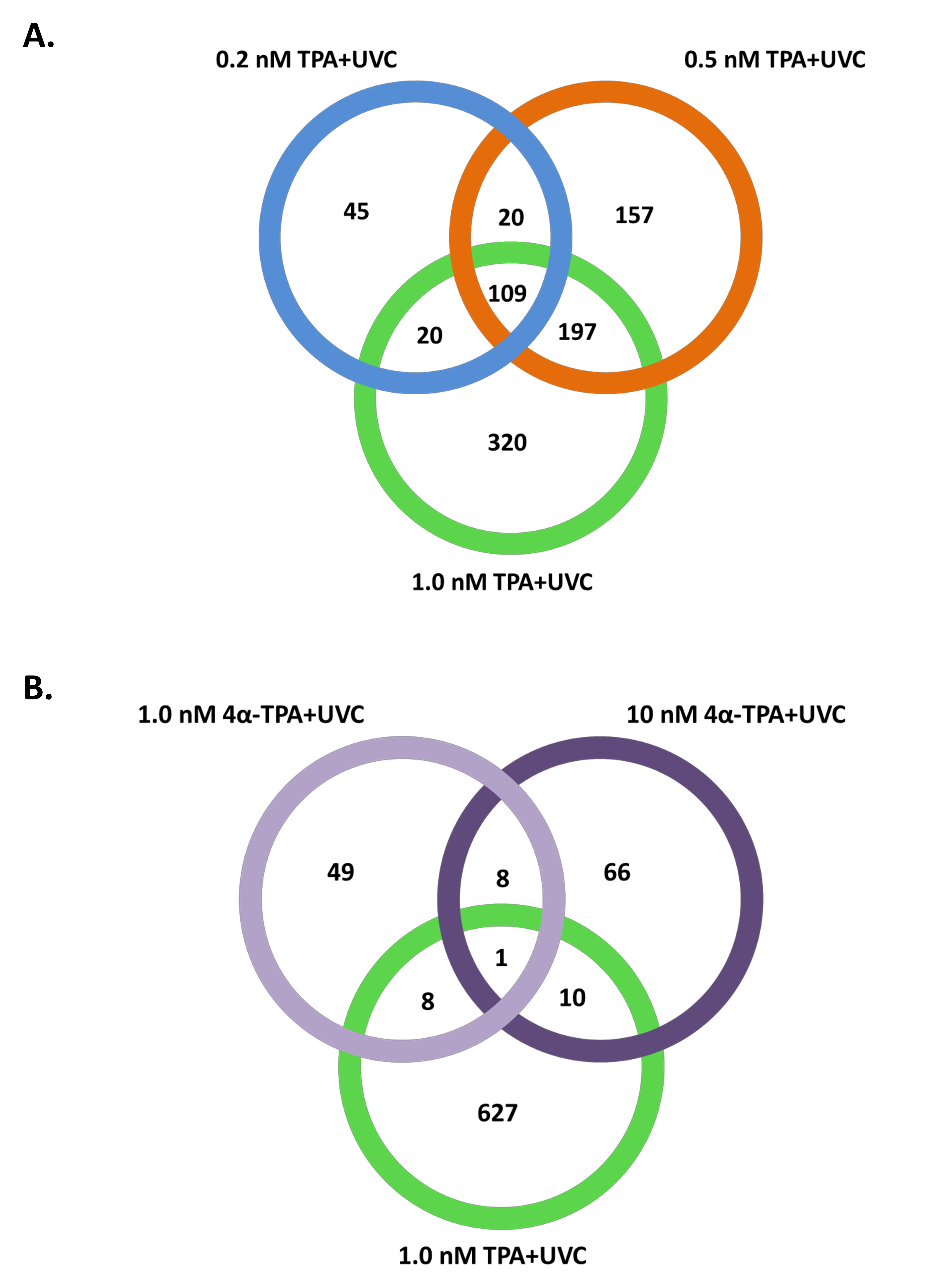

Supplement: S1 Fig — Differential expression (Fold-change ±2, FDR<0.05) was determined in the co-treated cells using the UVC-alone treatment as the control. Therefore, these genes were considered synergistically altered genes. (A) The number of SA-DEGs increased with the dose of TPA. Many of the 0.2+TPA SA-DEGs are also represented in the 0.5 nM and 1.0 nM TPA+UVC gene sets. (B) Little overlap of SA-DEGs was observed between two concentrations of the non-tumor promoting phorbol ester 4α-TPA+UVC and the 1.0 nM TPA+UVC. (TIF) [file pone.0139850.s001.tif]

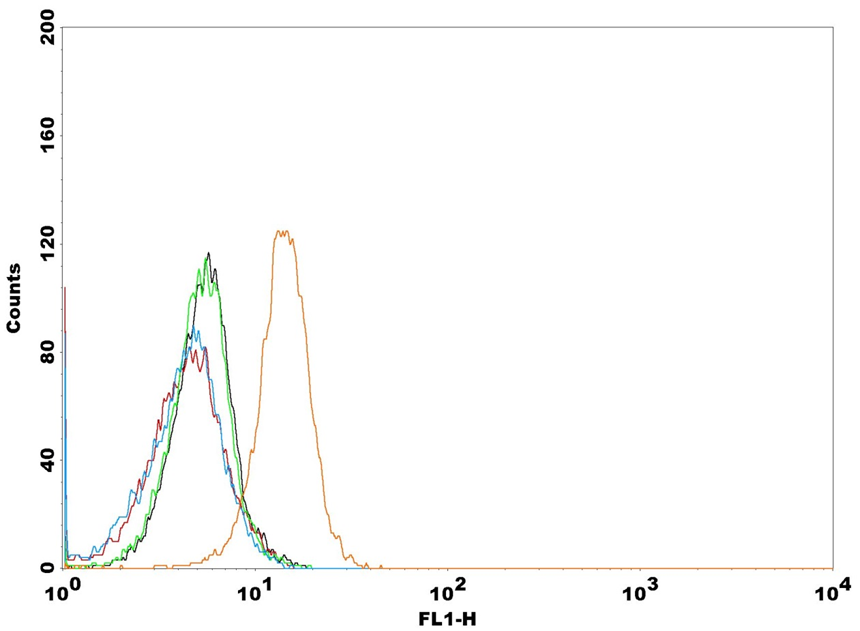

Supplement: S2 Fig — TK6 cells were analyzed at 1 hour after UVC-irradiation for increased ROS formation with a live cell oxidative stress probe (CellROX® Green Reagent, Life Technologies) using flow cytometry. Ten-thousand cells were analyzed per condition for untreated cells (black line), UVC-alone (green), TPA-alone (red) and TPA+UVC (blue). tert-Butyl hydroperoxide (TBHP) (orange) was used as a positive control. TPA-pretreated cells appeared to have less ROS based on a slight population shift in probe fluorescence. Other time points were also analyzed including 2, 4 and 8 hours post-irradiation with similar findings as the 1 hour time-point (data not shown). (TIF) [file pone.0139850.s002.tif]
