# Supplementary material for: Synergistic Gene Expression Signature Observed in TK6 Cells upon Co-Exposure to UVC-Irradiation and Protein Kinase C-Activating Tumor Promoters
Source: PLoS One. 2015 Oct 2;10(10):e0139850. doi: 10.1371/journal.pone.0139850 (PMC4592187; doi:10.1371/journal.pone.0139850)
Supplement: S1 Table — (DOCX) [file pone.0139850.s003.docx]

**S1 Table. Functional annotation summary of down-regulated genes by each treatment condition**

|  | **TPA** | |  | **UVC** | |  | **TPA+UVC** | |
| --- | --- | --- | --- | --- | --- | --- | --- | --- |
|  | *Cluster Summary* | *EASE* |  | *Cluster Summary* | *EASE* |  | *Cluster Summary* | *EASE* |
| **4-hr** | Inflammatory Response | 2.88 | **4-hr** | Phosphorylation | 10.49 | **4-hr** | Protein Catabolic Process | 9.24 |
|  | Circulation | 1.64 |  | Cell cycle/M phase | 8.91 |  | Cell Cycle | 7.06 |
|  | Response to nutrients | 1.45 |  | Protein catabolic process | 8.23 |  | Regulation of Transcription | 6.61 |
|  | Regulation of myeloid leukocyte immunity | 1.25 |  | Microtubule-based process | 7.34 |  | Chromatin Modification | 6.26 |
|  | Regulation of leukocyte immunity | 1.18 |  | Regulation of Ras GTPase pathway | 5.4 |  | Phosphorylation | 5.75 |
|  | Positive regulation of immune response | 1.15 |  | Transcription process | 4.83 |  | Protein Transport | 5.69 |
|  | Response to bacterium | 1.13 |  | Regulation of transcription | 4.79 |  | Microtubule-based Process | 5.27 |
|  | Humoral immune response | 1.1 |  | Protein localization | 4.49 |  | DNA damage response/repair | 3.9 |
|  | Regulation of cAMP signaling | 1.08 |  | Chromatin modification | 3.89 |  | Transcription Process | 3.77 |
|  | Response to radiation | 1.06 |  | Microtubule-based movement | 3.8 |  | Receptor Signaling | 3.64 |
| **8-hr** | Immune Response | 2.33 | **8-hr** | Phosphorylation | 3.85 | **8-hr** | Phosphorylation | 4.48 |
|  | Chromatin assembly | 1.45 |  | Regulation of Ras GTPase Pathway | 3.07 |  | Regulation of Ras GTPase signaling | 3.36 |
|  | Humoral immune response | 1.18 |  | Immune cell activation | 2.22 |  | Protein transport | 3.31 |
|  | Leukocyte activation | 1.15 |  | Cytoskeleton organization | 2.14 |  | Vesicle-mediated transport | 3.03 |
|  | Extracellular matrix organization | 1.08 |  | Glycosylation | 2.13 |  | Glycosylation | 2.6 |
|  | Glycosylation | 1.06 |  | Protein transport | 1.93 |  | Positive Regulation of apoptosis | 2.11 |
|  | Immune cell activation | 1.03 |  | Regulation of adhesion | 1.74 |  | Response to stimulus | 1.95 |
|  | Steroid metabolism | 1.03 |  | Microtubule-based transport | 1.64 |  | Chromatin modification | 1.87 |
|  | Protein complex assembly | 0.95 |  | Regulation of apoptosis | 1.38 |  | Regulation of cytoskeleton | 1.87 |
|  | G-protein signaling | 0.94 |  | Response to stimulus | 1.34 |  | Cell cycle/M phase | 1.8 |
| **24-hr** | Immune /inflammatory response | 2.04 | **24-hr** | Mitochondrial transport | 1.41 | **24-hr** | Steroid metabolism | 2.87 |
|  | Extracellular matrix organization | 1.43 |  | Positive regulation of immune effector | 1.13 |  | Carbohydrate metabolism | 1.8 |
|  | Response to nutrient | 1.16 |  | Ion homeostasis | 0.95 |  | Nucleotide metabolism | 1.58 |
|  | Regulation of protein assembly | 1.12 |  | Bone development | 0.91 |  | Immune Response | 1.51 |
|  | Vesicle organization | 1.08 |  | Ion transport | 0.85 |  | Chromatin assembly | 1.32 |
|  | Sensory organ development | 1.04 |  | Negative regulation of macromolecule metabolism | 0.56 |  | Ribonucleoside metabolism | 1.2 |
|  | Response to stimulus | 0.94 |  | Regulation of apoptosis | 0.53 |  | Response to stimulus | 1.12 |
|  | Glycosylation | 0.89 |  | Immune cell activation | 0.52 |  | Ion transport | 1.09 |
|  | Cardiac development | 0.82 |  | Response to stimulus | 0.5 |  | Response to drug/radiation | 0.97 |
|  | Ion homeostasis/chemotaxis | 0.82 |  | Protein Catabolic process | 0.32 |  | Positive regulation of cytokine production | 0.93 |
