# Supplementary material for: Synergistic Gene Expression Signature Observed in TK6 Cells upon Co-Exposure to UVC-Irradiation and Protein Kinase C-Activating Tumor Promoters
Source: PLoS One. 2015 Oct 2;10(10):e0139850. doi: 10.1371/journal.pone.0139850 (PMC4592187; doi:10.1371/journal.pone.0139850)
Supplement: S2 Table — (DOCX) [file pone.0139850.s004.docx]

**S2 Table. Functional annotation summary of up-regulated genes by each treatment condition**

|  | **TPA** | |  | **UVC** | |  | **TPA+UVC** | |
| --- | --- | --- | --- | --- | --- | --- | --- | --- |
|  | *Cluster Summary* | *EASE* |  | *Cluster Summary* | *EASE* |  | *Cluster Summary* | *EASE* |
| **4-hr** | Inflammatory response | 8.23 | **4-hr** | Response to extracellular stimulus | 2.25 | **4-hr** | Inflammatory Response | 4.06 |
|  | Positive regulation of apoptosis | 4.1 |  | Sensory perception | 1.5 |  | Cell Death | 3.36 |
|  | Apoptosis/Cell death | 3.16 |  | Negative regulation of growth | 1.44 |  | Positive Regulation of apoptosis | 2.35 |
|  | Immune cell differentiation | 2.91 |  | Cognition | 1.42 |  | Positive regulation of immune response | 2.2 |
|  | Immune system development | 2.71 |  | Response to oxidative stress | 1.4 |  | Response to stimulus | 1.9 |
|  | Immune cell activation | 2.44 |  | DNA damage response/apoptosis | 1.35 |  | Regulation of cytokine production | 1.66 |
|  | Chemotaxis/migration | 2.43 |  | Regulation of steroid biosynthesis | 1.27 |  | Epithelial differentiation | 1.59 |
|  | Response to stimulus | 2.32 |  | Epithelial differentiation | 1.24 |  | Positive regulation of immune cell activation | 1.55 |
|  | Response to LPS/bacterium | 2.31 |  | Response to stimulus | 1.24 |  | Response to oxidative stress | 1.41 |
|  | Response to oxidative stress | 2.23 |  | Inflammatory response | 1.18 |  | Regulation of cell growth | 1.34 |
| **8-hr** | Inflammatory response | 7.78 | **8-hr** | DNA damage response/apoptosis | 3.71 | **8-hr** | Inflammatory Response | 3.97 |
|  | Immune system development | 5.79 |  | Negative regulation of cell growth | 2.82 |  | Epithelial differentiation | 3.76 |
|  | Regulation of apoptosis | 5.62 |  | Regulation of apoptosis | 2.53 |  | Regulation of apoptosis | 3.55 |
|  | Immune cell activation | 4 |  | Regulation of kinase activity | 2.21 |  | Response to radiation/UV | 2.98 |
|  | Apoptosis | 3.75 |  | Response to UV/radiation | 2.21 |  | Regulation of apoptosis | 2.65 |
|  | Regulation of immune cell activation | 2.86 |  | Inflammatory response | 1.98 |  | DNA damage response/apoptosis | 2.33 |
|  | Lymphocyte activation | 2.5 |  | Response to stimulus | 1.61 |  | Regulation of immune cell activation | 2.15 |
|  | Chemotaxis | 2.31 |  | Epithelial differentiation | 1.51 |  | Ion homeostasis | 2.08 |
|  | Erythrocyte differentiation | 2.23 |  | Response to steroid hormone | 1.44 |  | Positive regulation of signal transduction | 1.75 |
|  | Regulation of signal transduction | 2.15 |  | Mesenchymal differentiation | 1.42 |  | Regulation of kinase activity | 1.67 |
| **24-hr** | Inflammatory response | 7.73 | **24-hr** | Epithelial cell development | 2.81 | **24-hr** | Inflammatory Response | 10.67 |
|  | Hemopoietic development | 3.33 |  | Inflammatory response | 2.78 |  | Regulation of immune cell activation | 5.89 |
|  | Regulation of cytokine production | 3 |  | Angiogenesis | 2.42 |  | Regulation of apoptosis | 5.74 |
|  | Response to LPS/bacterium | 2.74 |  | Lipid transport | 2.01 |  | Regulation of cytokine production | 5.15 |
|  | Regulation of protein transport | 2.71 |  | Regulation of apoptosis | 1.82 |  | Response to bacterium/LPS | 5.14 |
|  | Positive regulation of signal transduction | 2.68 |  | Collagen metabolism | 1.64 |  | Apoptosis/cell death | 4.34 |
|  | Chemotaxis | 2.63 |  | Negative regulation of growth | 1.58 |  | Cell migration | 3.88 |
|  | Positive regulation of apoptosis | 2.56 |  | Cell cycle process/arrest | 1.45 |  | Angiogenesis | 3.47 |
|  | Regulation of immune cell activation | 2.15 |  | DNA damage response and p53 | 1.42 |  | Chemotaxis | 3.03 |
|  | Regulation of myeloid differentiation | 1.96 |  | Positive regulation of signal transduction | 1.3 |  | Regulation of cytokine biosynthesis | 2.86 |
