# Supplementary material for: Synergistic Gene Expression Signature Observed in TK6 Cells upon Co-Exposure to UVC-Irradiation and Protein Kinase C-Activating Tumor Promoters
Source: PLoS One. 2015 Oct 2;10(10):e0139850. doi: 10.1371/journal.pone.0139850 (PMC4592187; doi:10.1371/journal.pone.0139850)
Supplement: S3 Table — (DOCX) [file pone.0139850.s005.docx]

**S3 Table. Number of genes in each conditions associated with GO:0043067 Regulation of Programmed Cell Death -**

|  | **UVC** | | | | **TPA** | | | | **TPA+UVC** | | | |
| --- | --- | --- | --- | --- | --- | --- | --- | --- | --- | --- | --- | --- |
| Time | **Count** | **%** | **P** | **FDR** | **Count** | **%** | **P** | **FDR** | **Count** | **%** | **P** | **FDR** |
| **4** | 135 | 5.6 | 2.10E-03 | 6.80E-02 | 54 | 8 | 4.80E-05 | 9.00E-03 | 174 | 5.6 | 4.50E-04 | 2.40E-02 |
| **8** | 120 | 6.4 | 1.30E-05 | 2.20E-02 | 48 | 9 | 3.20E-06 | 3.40E-04 | 191 | 6.2 | 2.70E-07 | 5.50E-04 |
| **24** | 54 | 8 | 1.70E-05 | 6.40E-03 | 40 | 8.1 | 5.50E-04 | 2.70E-02 | 129 | 7.5 | 1.50E-10 | 5.00E-08 |
