# Supplementary material for: Synergistic Gene Expression Signature Observed in TK6 Cells upon Co-Exposure to UVC-Irradiation and Protein Kinase C-Activating Tumor Promoters
Source: PLoS One. 2015 Oct 2;10(10):e0139850. doi: 10.1371/journal.pone.0139850 (PMC4592187; doi:10.1371/journal.pone.0139850)
Supplement: S4 Table — (DOCX) [file pone.0139850.s006.docx]

**S4 Table. Log transformed RPKtM values for each of the 17 key genes**

|  |  | Log2(RPKtM) | |  |  |
| --- | --- | --- | --- | --- | --- |
| gene | CCDS | MOCK_DMSO:8 | TPA:8 | UVC:8 | UVC_TPA:8 |
| TNC | CCDS6811.1\|Hs37.3\|chr9 | 0.0843 | 0.5131 | 0.7221 | 2.2488 |
| DNAH10 | CCDS9255.2\|Hs37.3\|chr12 | 0.1498 | 0.4259 | 1.2753 | 2.3655 |
| C3orf67 | CCDS33776.1\|Hs37.3\|chr3 | 0.869 | 0.86 | 2.1234 | 3.6568 |
| ZSCAN4 | CCDS12958.1\|Hs37.3\|chr19 | 0 | 0 | 2.3932 | 3.8017 |
| CCDC70 | CCDS9431.1\|Hs37.3\|chr13 | 0 | 0 | 1.313 | 4.2928 |
| IL29 | CCDS12531.1\|Hs37.3\|chr19 | 0 | 2.6265 | 0 | 4.2965 |
| SERPINE1 | CCDS5711.1\|Hs37.3\|chr7 | 0.3443 | 0.8118 | 3.7303 | 4.7362 |
| IFNG | CCDS8980.1\|Hs37.3\|chr12 | 1.6656 | 3.5232 | 2.207 | 5.3414 |
| FOS | CCDS9841.1\|Hs37.3\|chr14 | 4.0171 | 5.2567 | 5.4802 | 7.7689 |
| TNFSF4 | CCDS1306.1\|Hs37.3\|chr1 | 0.5729 | 6.7557 | 4.108 | 8.2662 |
| CSTA | CCDS3011.1\|Hs37.3\|chr3 | 5.2085 | 7.9164 | 7.4804 | 8.9302 |
| LIF | CCDS13872.1\|Hs37.3\|chr22 | 5.0088 | 5.9419 | 7.872 | 9.0169 |
| ATF3 | CCDS1506.1\|Hs37.3\|chr1 | 4.8772 | 6.6106 | 8.3651 | 9.419 |
| PPP1R15A | CCDS12738.1\|Hs37.3\|chr19 | 8.1393 | 9.4801 | 9.4505 | 10.5543 |
| GDF15 | CCDS12376.1\|Hs37.3\|chr19 | 7.045 | 10.1536 | 11.015 | 12.8083 |
| SAT1 | CCDS14207.1\|Hs37.3\|chrX | 9.0265 | 11.2665 | 11.2408 | 13.1397 |
| PMAIP1 | CCDS11975.1\|Hs37.3\|chr18 | 10.5122 | 11.2574 | 12.125 | 13.2407 |
